# Population Genetic Structure and Contribution of Philippine Chickens to the Pacific Chicken Diversity Inferred From Mitochondrial DNA

**DOI:** 10.3389/fgene.2021.698401

**Published:** 2021-07-22

**Authors:** Cyrill John P. Godinez, Peter June D. Dadios, Dinah M. Espina, Megumi Matsunaga, Masahide Nishibori

**Affiliations:** ^1^Laboratory of Animal Genetics, Graduate School of Integrated Sciences for Life, Hiroshima University, Higashihiroshima, Japan; ^2^Department of Animal Science, College of Agriculture and Food Science, Visayas State University, Baybay City, Philippines; ^3^College of Aquatic and Applied Life Sciences, Southern Leyte State University, Southern Leyte, Philippines

**Keywords:** demographic history, *Gallus gallus*, genetic structure, mtDNA, Philippine chickens, phylogeography

## Abstract

The Philippines is considered one of the biodiversity hotspots for animal genetic resources. In spite of this, population genetic structure, genetic diversity, and past population history of Philippine chickens are not well studied. In this study, phylogeny reconstruction and estimation of population genetic structure were based on 107 newly generated mitochondrial DNA (mtDNA) complete D-loop sequences and 37 previously published sequences of Philippine chickens, consisting of 34 haplotypes. Philippine chickens showed high haplotypic diversity (*Hd* = 0.915 ± 0.011) across Southeast Asia and Oceania. The phylogenetic analysis and median-joining (MJ) network revealed predominant maternal lineage haplogroup D classified throughout the population, while support for Philippine–Pacific subclade was evident, suggesting a Philippine origin of Pacific chickens. Here, we observed Philippine red junglefowls (RJFs) at the basal position of the tree within haplogroup D indicating an earlier introduction into the Philippines potentially via mainland Southeast Asia (MSEA). Another observation was the significantly low genetic differentiation and high rate of gene flow of Philippine chickens into Pacific chicken population. The negative Tajima’s *D* and Fu’s *Fs* neutrality tests revealed that Philippine chickens exhibited an expansion signal. The analyses of mismatch distribution and neutrality tests were consistent with the presence of weak phylogeographic structuring and evident population growth of Philippine chickens (haplogroup D) in the islands of Southeast Asia (ISEA). Furthermore, the Bayesian skyline plot (BSP) analysis showed an increase in the effective population size of Philippine chickens, relating with human settlement, and expansion events. The high level of genetic variability of Philippine chickens demonstrates conservation significance, thus, must be explored in the future.

## Introduction

The rich history of human migrations and settlements in the islands of Southeast Asia (ISEA) provides interesting records of earlier agricultural populations in the Malay Archipelago (Bellwood, 2007; Piper, 2017). The two-wave hypothesis of peopling in the ISEA provides different interpretations of the prehistoric evolution of the indigenous populations in the insular (Jinam et al., 2012). The mid-Holocene human migration epoch in the ISEA was believed to have brought varieties of material culture, initial farming communities for rice agriculture, and domestic animals particularly, dogs, pigs, and chickens (Diamond and Bellwood, 2003; Piper, 2017). Archeological and linguistic evidence documented the movement of Taiwan-centered Austronesian speakers to the Northern Philippines estimated at 4,000 *cal.* BP, then widespread to the south and west into the ISEA toward Indonesia and east into the Pacific Islands at *ca.* 3,300–3,150 *cal.* BP (Bellwood and Dizon, 2013). Recently, genetic data documented both Taiwan-centered Austronesian expansion and an earlier introduction from mainland Southeast Asia (MSEA) to the insular, predating the mid-Holocene human migration model (Jinam et al., 2012; Lipson et al., 2014; Soares et al., 2016; Arenas et al., 2020). Evidence of diverse migration routes and dispersal events documented rapid human population expansion toward the Philippines (Arenas et al., 2020). These human-mediated scenarios linking domestic animal translocations present wide interests in understanding the chicken domestication events in the ISEA.

The Philippines is considered one of the most biologically rich regions in the world in terms of animal genetic resources and one of the leading biodiversity hotspots in the Indo-Australian archipelago based on animal endemism per area ratios (Myers et al., 2000). However, there is insufficient evidence that links the present-day chickens to their ancient lineages due to unclear timeline of translocations and routes of dispersal across ISEA. Unlike chickens, the distribution of domestic pigs corresponds to the proposed origins and expansion of the Pacific clade pigs from Southern China and across parts of MSEA, following the movement of Austroasiatic speakers (Larson et al., 2007; Piper et al., 2014). Despite limited Neolithic zooarchaeological records of Philippine chickens (Piper, 2017), ancient DNA recovered from Pacific chickens documented potential traces of origin from the Philippines (Thomson et al., 2014). Though still enigmatic, no direct evidence of domestic chickens’ introduction to the Philippines prior to 4,500 *cal.* BP can be found. Most likely, the multiple wave of human translocations exerted a huge influence on the earlier lineages of domestic chickens introduced in the Philippines (Jinam et al., 2012; Piper, 2017).

The domestication of chickens has contributed various benefits to the sustenance and cultural development of mankind. The profound history of chicken domestication has attracted wide interest in molecular phylogeny and phylogeographic patterns as it remains debatable up to today. Previous findings on reconstruction of the matrilineal lineages of domestic chickens documented that the red junglefowl (RJF) from MSEA served as the progenitor of all present-day chickens (Fumihito et al., 1994, 1996). However, molecular evidence of multiple matrilines has suggested independent domestication events across Asia and the Indian subcontinent, supporting multiple origins of domestic chickens (Nishibori et al., 2005; Liu et al., 2006; Kanginakudru et al., 2008).

Therefore, in the present study, complete mitochondrial DNA (mtDNA) D-loop sequences from RJFs and native chickens (NCs) in the Philippines were investigated to assess their matrilineal phylogeny, genetic diversity, and population genetic structure of Philippine chickens across ISEA and the Pacific. In addition, this study attempted to reconstruct the population history and probable dispersal of Philippine chickens throughout the Pacific.

## Materials and Methods

### Sample Collection

Blood samples were collected from the brachial vein of the wing of RJFs (*n* = 7) and NCs (*n* = 100) from selected areas of Samar and Leyte Provinces, Philippines. RJFs were captured in the wild by the locals. All samples were collected following the Experimental Animal Care Guidelines established by the Laboratory of Animal Genetics, Hiroshima University (015A170426). Animal owners consented the inclusion of their animals in the study.

### DNA Extraction, Amplification, and Sequencing

Genomic DNA was extracted from stored whole blood samples of Philippine RJFs and NCs using the phenol–chloroform method following the recommended protocol described by Green et al. (2012).

About 5.0-kbp mtDNA D-loop fragments were amplified using a long and accurate PCR (LA-PCR) kit (KOD-FX Neo Polymerase, Toyobo, Osaka, Japan) with chicken DNA as a template and LA-PCR primer sets: *Cytb-Forward*: 5′-TACACG AATCAGGCTCAAACAACCCCCTAGGCATC-3′, *16S-Reverse*: 5′-TGCACCATTAGGTTGTCCTGATCCAACATCGAGGT-3′ recommended by Nishibori et al. (2003). The reaction began with a preliminary denaturation at 94°C for 2 min, followed by 30 cycles of DNA denaturation at 98°C for 10 s, annealing of primers at 57°C for 30 s, and primer extension at 68°C for 2 min and 30 s, using a GeneAmp PCR System 9700 (Applied Biosystems, Foster City, CA, United States). Amplified fragments were used for segmental amplification of the complete mtDNA D-loop region (1.3 kbp) following the primer sets *Gal1F* 5′-AGGACTACGGCTTGAAAAGCCATTG-3′ and *Gal1R* 5′-GCTGAGTACCCGTGGGGGTGTGGCT-3′ in a 20-μl reaction volume containing 2 × PCR buffer, 0.4 mM dNTPs, 0.3 μM concentrations of each primers, 0.4 U of KOD-FX Neo DNA Polymerase, and 15–25 ng of amplified fragment DNA as template. The PCR cycling condition began with a preliminary denaturation at 94°C for 2 min, followed by 30 cycles of DNA denaturation at 98°C for 10 s, annealing of primers at 59°C for 30 s, and primer extension at 68°C for 30 s, using a GeneAmp PCR System 9700 (Applied Biosystems, Foster City, CA, United States). The DNA fragments obtained from the segmental amplification were cleaned and purified using Exonuclease I (ExoI) and Shrimp Alkaline Phosphatase (SAP) to degrade the residual PCR primers and dephosphorylate the remaining dNTPs, respectively. Subsequently, the mtDNA D-loop fragments were directly sequenced using 3130/3130xl Genetic Analyzers (Applied Biosystems, Foster City, CA, United States).

### DNA Sequence Alignment

The 107 complete mtDNA D-loop sequences generated in this study were edited initially using GeneStudio Pro tool (GeneStudio, Inc.)^1^ and were aligned together with 495 complete mtDNA D-loop sequences across Asia using ClustalW (Thompson et al., 1994). Previous sequences of Samar RJFs (*n* = 3) (MK085033–MK085035) and Samar NCs (*n* = 17) (MK085038–MK085054) (Godinez et al., 2019) and other Philippine chicken complete D-loop sequences retrieved from GenBank (*n* = 17) were also included in the analysis (Supplementary Table 1). Aligned nucleotide sequences (corresponding to the chicken mtDNA reference sequence, accession no. NC_040970) were edited and viewed using the BioEdit sequence alignment editor (Hall, 1999). All complete mtDNA D-loop sequences of RJFs and representative sequence from identified haplotypes of Philippine NCs were deposited in the GenBank database with accession numbers MN986370–MN986403 (Supplementary Table 1).

### Genetic Diversity and Phylogenetic Reconstruction

Intrapopulation-level genetic diversity indices, such as the number of polymorphic (segregating) sites (S), haplotype diversity (*Hd*), nucleotide diversity (π), and mean number of pairwise difference, were estimated using the DnaSP v. 6.0 software (Librado and Rozas, 2009).

Phylogenetic tree was inferred by IQ-TREE using the maximum likelihood (ML) method (Nguyen et al., 2015) to estimate the genealogy of Philippine chickens together with the other complete mtDNA D-loop sequences from Indonesian and Pacific chickens, and other sequences of RJFs and NCs across Asia retrieved from GenBank (Supplementary Table 1). The best-fit substitution model was determined based on the Bayesian information criterion using jModeltest v2.1.10 (Darriba et al., 2012). Node support was estimated using 1,000 ultrafast bootstrap replicates (Hoang et al., 2018). The nomenclatures of the 13 haplogroups (haplogroups A to I and haplogroups W to Z) reported by Miao et al. (2013) and haplogroup V (Huang et al., 2018) were used as references for the haplogroup notations. The list of haplotypes used and the corresponding GenBank accession numbers are provided in the Supplementary Table 1. Median-joining (MJ) network was constructed to infer the evolutionary relationships among chicken haplotypes using the NETWORK 4.6 software (Bandelt et al., 1999). This method calculates the net divergence of each taxon from all other taxa as the sum of the individual distances from variance within and among groups. The number and assignment of haplotypes were determined using the DnaSP v. 6.0 software.

Truncated partial sequences (764-bp fragment) were also analyzed for more fine-grained phylogeographic analysis of chicken population in the ISEA and Pacific region together with other partial sequences (Supplementary Table 3) from Indonesian and Pacific chickens (Dancause et al., 2011; Thomson et al., 2014). Bootstrap values were estimated with 1,000 repetitions.

### Population Genetic Structure and Demographic History

The population pairwise net genetic distance based on population pairwise *F*_ST_ (significant values were accepted at *p* < 0.05) and Slatkin’s linearized *F*_ST_ was estimated using the Arlequin v. 3.5.2.2 software (with 10,000 permutation) (Excoffier et al., 2005). The level of significance was evaluated based on 1,023 random permutations. To visualize the pattern of genetic relationship among the populations, the haplotypic pairwise differences were plotted into principal coordinate analysis (PCoA) using GenAlEx v. 6.503. To further estimate the genetic structure of each population among geographic groups, analysis of molecular variance was performed as implemented by the Arlequin v. 3.5.2.2 software. Significance testing was evaluated using 10,000 coalescent simulations.

Past demographic parameters were inferred by the analysis of the distribution of the number of site differences (mismatch distribution) using the program DnaSP v. 6.0 software (Librado and Rozas, 2009). Expected (simulated) values under expanding population model were calculated and plotted against the observed values. Populations that have undergone recent demographic growth tend to show a unimodal distribution without large differences in the frequency of the ranked pairwise differences, while those populations at demographic equilibrium present a multimodal distribution (Rogers and Harpending, 1992). Raggedness statistics, *r* (Harpending, 1994), was used to quantify the smoothness of the mismatch distributions, and the confidence intervals were provided by coalescent algorithm simulations using the DnaSP v. 6.0 software. The sum of squared deviations (SSD), as implemented in Arlequin v. 3.5.2.2, was used to further evaluate the sudden expansion model (Rogers and Harpending, 1992; Rogers, 1995). To further support the inference for population growth model, we used more powerful neutrality statistical tests, such as Tajima’s *D* (Tajima, 1989) and Fu’s *Fs* statistics (Fu, 1997). These population expansion tests measure haplotype frequencies under neutrality and panmixis. Statistical tests and confidence intervals were based on coalescent simulation algorithm under a neutral infinite-site model.

The coalescent-based methods had been widely used to quantify the relationship between the genealogy of the sequences and the demographic history of the population. The Bayesian skyline plot (BSP) (Drummond et al., 2005) was estimated to infer deeper insight on the demographic history of Philippine chickens as implemented in BEAST v. 2.6.3 (Bouckaert et al., 2019). The BSP was generated with a strict molecular clock model and setting with 3.13 × 10^−7^ mutations/site/year rate (Alexander et al., 2015). The piecewise constant function and HKY nucleotide substitution model was used for the analysis. The Markov chain Monte Carlo (MCMC) chain was run for 5 ×10^7^ generations, with a sampling of parameters every 5,000 steps and 5 ×10^6^ generations served as burn-in. Convergence of the posterior estimates of the effective population size (*N*_*e*_) to the likelihood stationary distribution was evaluated using the Tracer v. 1.7.1 software (Rambaut et al., 2018).

## Results

### Mitochondrial DNA Variation and Genetic Diversity

A total of 144 complete mtDNA D-loop sequences (1,232 bp) of Philippine chickens were analyzed in this study of which 107 were newly generated. There were 34 haplotypes (18 parsimony-informative sites) identified, 29 of which were possessed by NCs with 1 haplotype (Hap_68) shared with a RJF, while 5 haplotypes are unique in the RJF samples. The overall haplotypes of Philippine chickens (RJFs and NCs) had 32 polymorphic sites (all transition substitutions). The distribution of the nucleotide positions and sequence variations of each haplotype are presented in Supplementary Table 2.

The haplotype (gene) diversity (*Hd*) was relatively high ranging from 0.884 ± 0.103 in RJFs to 0.904 ± 0.012 in NCs (overall; *Hd* = 0.915 ± 0.051). The overall gene diversity concurred with the previously described intrapopulation genetic diversity of chicken populations in Samar, Philippines (Godinez et al., 2019). Nucleotide diversity (π) varied between 0.0017 ± 0.0003 and 0.0044 ± 0.0002 in RJFs and NCs, respectively (overall; π = 0.0043 ± 0.0002). The total mean number of pairwise difference was 4.256 ± 2.358, with the higher value observed among Philippine NCs (Table 1).

**TABLE 1:**
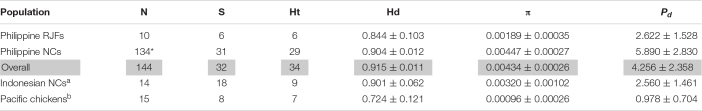
Genetic diversity indices of red junglefowl (RJF) and native chicken (NC) populations [complete mitochondrial DNA (mtDNA) D-loop sequence] and their haplogroup distributions.

*Included 17 sequences derived from Genbank (Herrera et al., 2018 – direct submission).

*^*a,b*^Sequence data from Herrera et al. (2018) – direct submission.*

*N, number of sequences; *S*, number of polymorphic (segregating) sites; *Ht*, number of haplotypes; *Hd*, haplotype (gene) diversity; π, nucleotide diversity; *P*_*d*_, mean number of pairwise difference.*

*Italic values indicate combined values for Philippine RJFs and Philippines NCs (all samples).*

### Phylogeography and Distribution of Philippine Chicken Haplogroups

**FIGURE 1:**
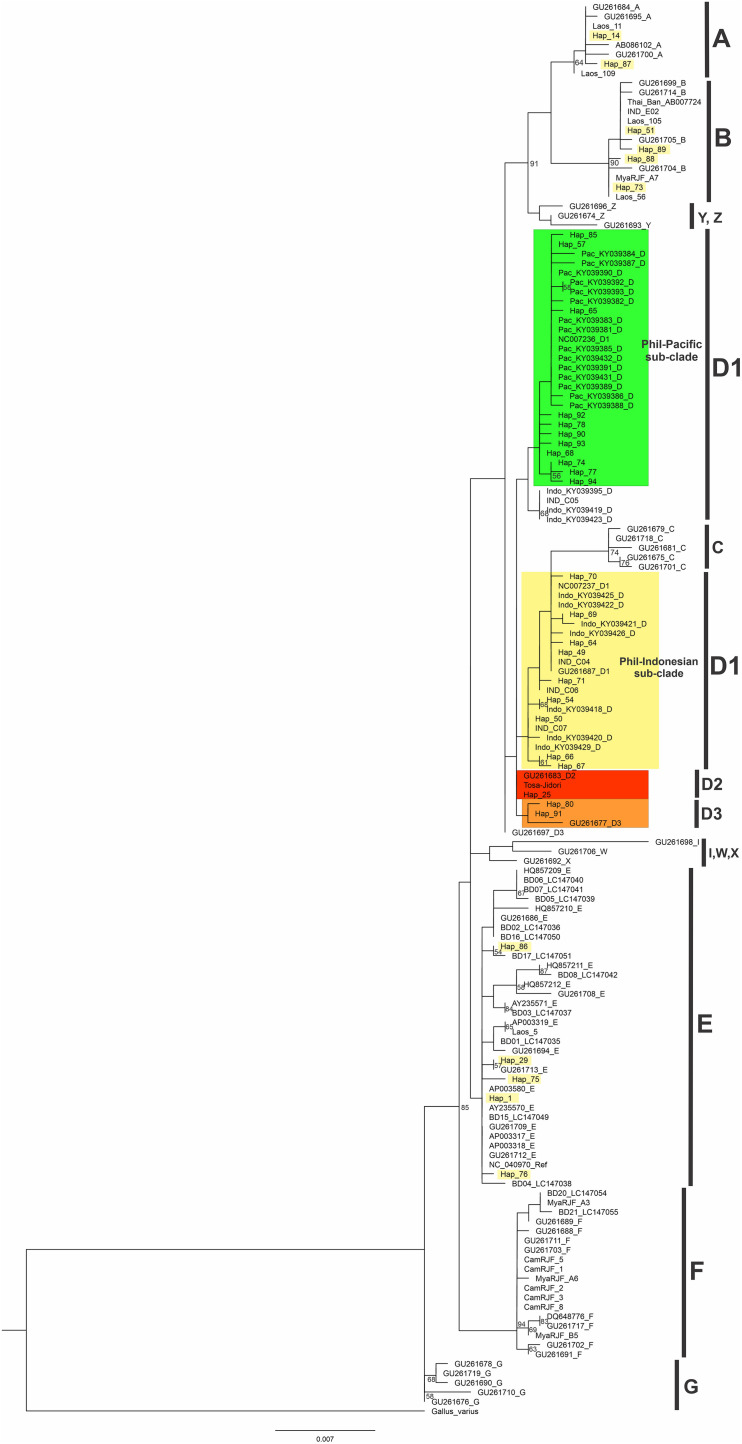
Maximum likelihood phylogenetic tree for complete mtDNA D-loop nucleotide sequences of Philippine chickens. Node labels correspond to bootstrap support values evaluated with 1,000 ultrafast bootstrap replicates in IQ-TREE. The scale bar (0.007) indicates the genetic distance (substitution per site). Bootstrap values under 50% are not shown.

The phylogenetic analysis of Philippine chickens together with the other chicken populations in the Pacific, Indonesia, MSEA, and sequences derived from the global mtDNA phylogeographic study (Miao et al., 2013; Huang et al., 2018) were investigated. Hypervariable region (HVR) was also analyzed to accommodate other D-loop partial sequences (754 bp) for a finer phylogeographic analysis of chicken populations in the ISEA and Pacific region (Supplementary Table 3). The rooted ML tree revealed four haplogroups (A, B, D, and E) of Philippine chickens. The majority (70.13%) of the samples using the complete mtDNA D-loop region belonged to haplogroup D with 23 haplotypes and the rest belongs to haplogroups A, B, and E (Figure 1 and Supplementary Figure 1). It was found that the Philippine RJFs clustered to haplogroup D. There were four RJF samples (i.e., MN986398–MN986400, MN986403) in Hap_25 unprecedentedly clustered to subhaplogroup D2 together with the fighting cock Tulufan from Xinjiang, China (as the reference subhaplogroup D2 nomenclature) (Miao et al., 2013) and Tosa-Jidori from Japan (Oka et al., 2007), which is reported to be related to Philippine RJFs for the first time in the present study (Figure 1 and Supplementary Figure 1). The other three RJFs were unclassified to any previously identified subhaplogroup nomenclatures for haplogroup D as patterned from the global profile (Miao et al., 2013) but appeared to be a sister group to the subhaplogroup D3 or subhaplogroup D3b (mutational motif T220C; A281G) (Huang et al., 2018; Figure 1 and Supplementary Figure 1). The Philippine NCs with predominant haplogroup D showed close genetic relationship to the Pacific and Indonesian chickens compared with chicken populations from the MSEA. Within haplogroup D, the rooted ML tree interestingly revealed a subclade for Philippine–Pacific chickens and another subclade for Philippine-Indonesian chickens (Figure 1).

**FIGURE 2:**
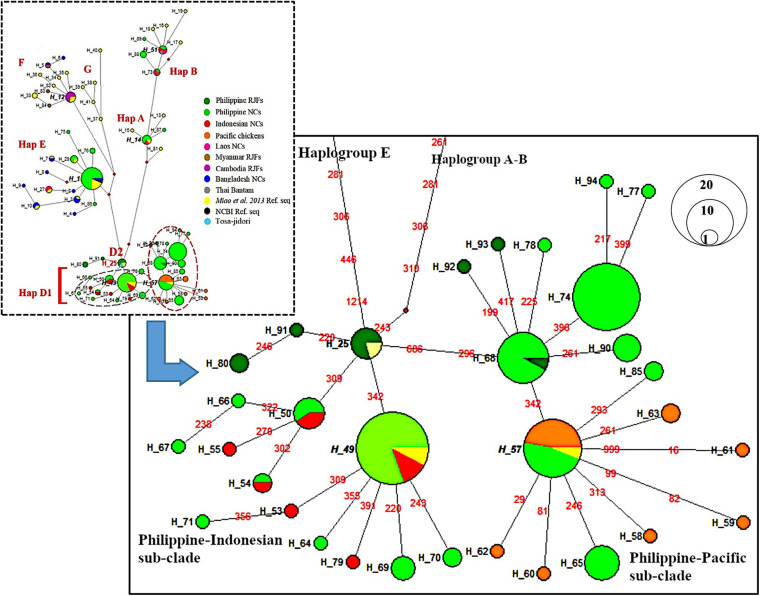
Median-joining network of the complete mtDNA D-loop region (1,232 bp) depicting relationship of Philippine chickens, Indonesian chickens, and Pacific chickens. The area of each circle is proportional to the frequency of the corresponding haplotypes. The length of branch connecting to other haplotypes correspond to mutational positions.

The MJ network further revealed consistent distinction of four maternal haplogroups (A, B, D, and E) of Philippine chickens (Figure 2). Clearly, within haplogroup D, there are two dominant haplotypes that distinguished the two subclades between Philippine–Pacific chickens (H_57) and Philippine–Indonesian chickens (H_49). Haplotype H_57 was grouped with Philippine RJF (NC_007236) (Nishibori et al., 2005; Miao et al., 2013) along with other samples of Philippine NCs and Pacific chickens. The genetic distance clarified two unprecedented mutation signatures of the Philippine–Pacific subclade with transition substitutions at the nucleotide positions C296T and G686A, while diverging to the Philippine–Indonesian subclade with the absence of those identified mutational motif (Supplementary Table 2). These findings agreed with the previously defined diagnostic motif (SNPs A281G, C296T, T306C, and A342G) from the ancient Pacific chicken sequences relative to the Philippine chickens (Thomson et al., 2014). However, the present study accounted for the complete mtDNA D-loop sequences and found diagnostic motif of SNPs at the 686-nucleotide position. Furthermore, a wider analysis of haplogroup D using HVR (*n* = 849) consistently showed distinct subclades of these chicken populations in the ISEA and Pacific region (Supplementary Figure 2).

### Population Genetic Structure and Expansion

The previous mtDNA study on Philippine chickens was limited only to the matrilineal phylogenetic analyses (Godinez et al., 2019). Here, we calculated the population genetic differentiation using pairwise divergence (*F*_ST_), Slatkin’s linearized *F*_ST_, and pairwise differences among populations of Philippine chickens (RJFs and NCs), Pacific chickens, Indonesian chickens, and MSEA chickens. Low genetic differentiation was observed between Philippine and Indonesian chickens (pairwise *F*_ST_; 0.1540 and Slatkin’s *F*_ST_; 0.1821) and between Philippine and Pacific chickens (pairwise *F*_ST_; 0.2681 and Slatkin’s *F*_ST_; 0.3663), which suggest that chicken populations in these regions were not isolated from each other (Table 2). The *F*_ST_ values between Philippine and Indonesian chickens (*F*_ST_ = 0.1540; 0.1821) were lower than Philippine and Pacific chickens (*F*_ST_ = 0.2681; 0.3663), which suggest a genetic closeness in the former populations due to geographical proximity and closer maritime ranges. However, interesting findings documented high genetic divergence (*F*_ST_ = 0.4788) between Pacific and Indonesian chickens, while chickens from the MSEA were the most remotely related to the Pacific chickens (*F*_ST_ = 0.5916). The PCoA analysis support clustering of closely related Philippine and Pacific chickens within haplogroup D, while distant to Indonesian chickens (Supplementary Figure 3). Both the *F*_ST_ values and population pairwise differences are consistent with previous analysis that showed the Philippines as potentially the key contributor of the diversity and genetic characteristics of Pacific chickens. All the *F*_ST_ values and population pairwise comparisons were significant at the 5% level.

**TABLE 2:** Genetic divergence between populations of Philippine chickens (RJFs and NCs), Indonesian, Pacific, and mainland Southeast Asia (MSEA) chickens at complete mtDNA D-loop sequences.

**(a) Population pairwise *F*_ST_ and Slatkin’s linearized *F*_ST_ (Population)**	**(1)**	**(2)**	**(3)**	**(4)**
(1) Philippine chickens^a^		0.1821	0.3663	0.6076
(2) Indonesian native chicken^b^	0.1540**		0.9188	0.6695
(3) Pacific chickens^b^	0.2681**	0.4788**		1.4487
(4) MSEA chickens^c^	0.3779**	0.4010**	0.5916**	
(b) Population average pairwise differences				
(1) Philippine chickens^a^		6.1231*	5.3178*	11.0679**
(2) Indonesian native chicken^b^	1.0373		5.1777**	12.1250**
(3) Pacific chickens^b^	2.0207	2.3606		13.1833**
(4) MSEA chickens^c^	3.5101	5.0469	7.8940	

^*a*^RJFs and NCs combined populations.

*Significant *F*_*ST*_ at *p* < 0.05.

**Highly significant *F*_*ST*_ at *p* < 0.001.

*^*b*^Direct submission sequences retrieved from Genbank.*

*^*c*^Sequences from Osman and Nishibori (2014). (a) Population pairwise genetic distance. Lower triangular matrix, population pairwise estimates of *F*_*ST*_; upper triangular matrix, slatkin’s Linearized *F*_*ST*_. (b) Population average pairwise genetic differences. Upper triangular matrix, average number of pairwise differences between populations (PiXY); lower triangular matrix, corrected average pairwise difference [PiXY - (PiX + PiY)/2].*

The analysis of molecular variance supports the low genetic differentiation of chicken populations in the ISEA with 79.18% of genetic variation showing significance within populations. Consistently, Philippine–Indonesian (i.e., Group B) and Philippine–Pacific (i.e., Group C) showed lower among-group variances with 9.43 and 20.97%, respectively, while higher among-group variance is observed in the Philippine–MSEA (i.e., Group D) with 34.17% (Table 3). However, the analysis showed higher genetic differentiation within the Pacific-Indonesian group (55.05% within-population variance, *p* = 0.000) than within the Philippine–Pacific group (69.61% within-population variance, *p* = 0.000) at haplogroup D (Table 3).

**TABLE 3:**
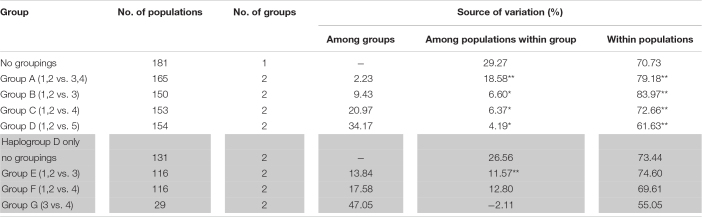
Population genetic structure estimated from the AMOVA based on complete mtDNA D-loop sequences from (1) Philippine RJFs, (2) Philippine NCs, (3) Indonesian NCs, (4) Pacific chickens, and (5) MSEA chickens.

*Significant *F*_*ST*_ at *p* < 0.05. **Significant *F*_*ST*_ at *p* < 0.01. Gray-shaded area corresponds to analysis for haplogroup D only.

**FIGURE 3:**
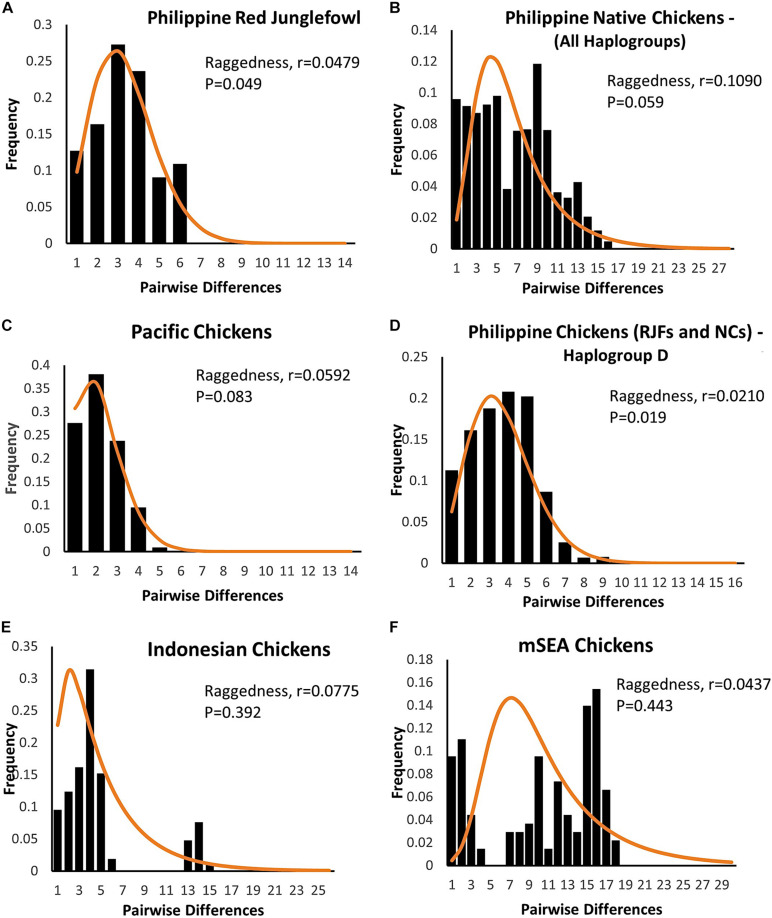
Mismatch distribution of the complete mtDNA D-loop sequences of **(A,B,D)** Philippine chickens [red junglefowls (RJFs) and NCs], **(C)** Pacific, **(E)** Indonesian, and **(F)** mainland Southeast Asian (SEA) chickens based on pairwise nucleotide site differences. The solid line indicates the theoretical distribution under population expansion model. The raggedness statistics and corresponding *p-*values for **(A)** Philippine RJFs; *r* = 0.0479, *p* = 0.049 and **(D)** RJFs–NCs–haplogroup D; *r* = 0.0210, *p* = 0.019, provided statistical support for the smoothness of the observed distributions.

The mismatch distributions of Philippine chickens (haplogroup D), Philippine RJFs, and Pacific chickens were unimodal (Figure 3), characteristic of a population that have undergone expansion. Support for the smoothness of the observed distributions was statistically fit for Philippine chickens haplogroup D and RJFs as quantified by raggedness statistics and coalescent algorithm simulations. In agreement, the observed distributions from all populations did not significantly deviate (SSD values > 5%) from the simulated values under the assumption of population expansion (Table 4). However, Philippine NCs (including all haplogroups in the dataset), along with the Indonesian chickens and MSEA chickens, exhibited a ragged mismatch distribution with high raggedness statistics (*r*) values. Both Tajima’s *D* and Fu’s *Fs* neutrality tests further indicated that chickens from the ISEA and Pacific region deviated from neutrality except chickens from MSEA, which support a model of demographic expansion. The negative and significant *Fs* statistical values in Philippine chickens (haplogroup D) and Pacific chickens provided strong evidence of population growth signatures of these chicken populations in the region (Table 4). Evidence for an excess of recent mutations and/or rare nucleotide site variants has been observed in the Philippine chickens considering all other haplogroups A, B, and E and Indonesian chickens under selective neutrality model, but the excess was statistically nonsignificant (Fu, 1997).

**TABLE 4:**
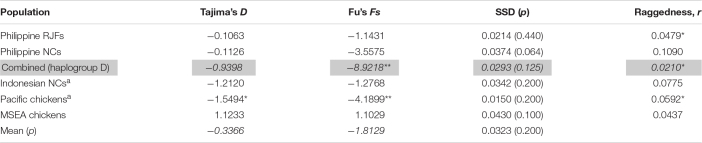
Neutrality tests and mismatch analysis sums of squared deviation (SSD) and Harpending’s raggedness index for Philippine RJF and NC complete mtDNA D-loop sequence.

*p-value < 0.05; **p-value < 0.01.

*^*a*^Direct submission sequences retrieved from GenBank.*

*Italic values indicate combined values for Philippine RJFs and Philippine NCs (using Haplogroup D only).*

**FIGURE 4:**
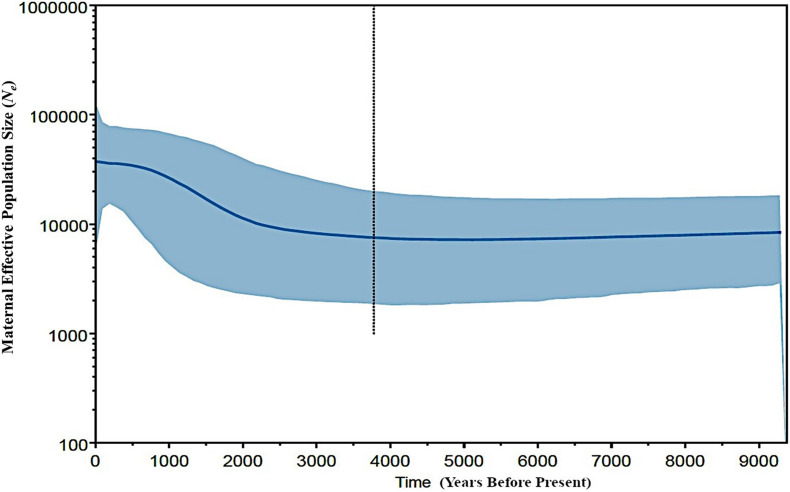
Bayesian coalescent skyline plot showing estimated demographic history of Philippine chickens (haplogroup D). The central blue line is the median estimate effective population size. The shaded area shows the upper and lower estimates of 95% credibility interval. The vertical dotted line represents the median estimate of time to the most recent common ancestor. The *x*-axis is the time (in years before present), and the *y*-axis indicates population size (as the product of *N*_*e*_ and the generation length in years).

**FIGURE 5:**
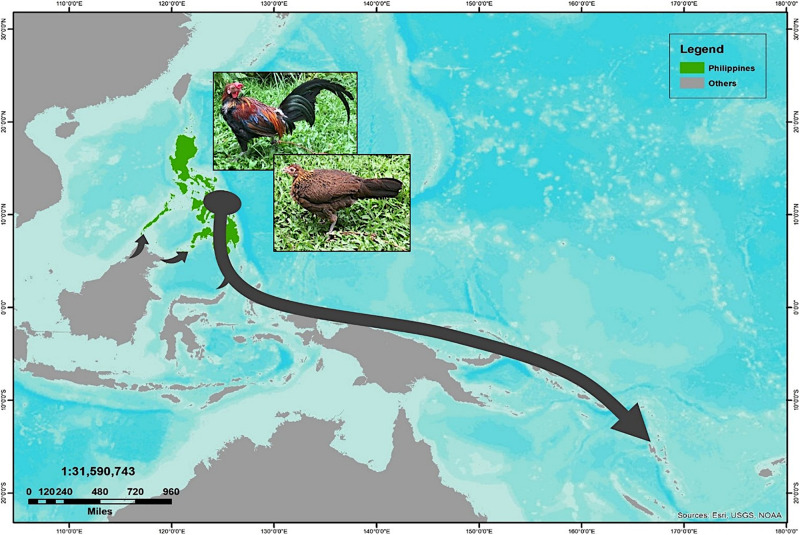
The proposed routes of translocation scenario of Philippine chickens expanding to the Pacific relating to the Austronesian speakers movement, as supported by the population pairwise genetic divergence (*F*_ST_) estimates, mismatch distribution analyses, and demographic inference based on coalescent simulation of Bayesian skyline plot (BSP).

In an attempt to obtain a better inference for the demographic history of the Philippine chickens, we evaluated the changes in maternal effective population sizes (*N*_e_) at different points along the genealogical timescale. The BSP showed evidence of Philippine chickens experiencing a long period of relatively constant *N*_e_ during the early Holocene period, followed with a gradual increase, which started approximately 3,500 BP, while an episode of eminent population growth commenced about 3,000 BP (Figure 4). Taken together, our analyses of population pairwise *F*_ST_, mismatch distributions, and BSP were consistent in showing that the Philippines was the main contributor to the diversity and genetic characteristics of Pacific chickens (Figure 5), related to the eastward movement of the Austronesian speakers from the Philippines approximately 3,000 years ago (kya) (Bellwood, 2007; Soares et al., 2016).

## Discussion

Philippine chickens have been shown to have high haplotypic diversity with a relatively large proportion of low-frequency haplotypes in the predominant haplogroup D (gene diversity = 0.915 ± 0.011). They show higher genetic diversity than Indonesian crowing chickens (Ulfah et al., 2017), Thai indigenous chickens (Teinlek et al., 2018), Laotian (Kawabe et al., 2014), and Vietnamese chickens (Cuc et al., 2011), North African NCs (e.g., Osman et al., 2016), West Africa NCs (e.g., Adebambo et al., 2010), Central Africa chickens (Hassaballah et al., 2015), and all East African chickens combined (Mwacharo et al., 2011), except Chinese chickens (*Hd* = 0.916; π = 0.00591) (Gao et al., 2017; Guo et al., 2017). The high genetic diversity in Philippine chickens resulted from the presence of abundant haplotype signatures (13 parsimony-informative sites and 10 singletons) in the predominant haplogroup D, which points that the population is large and expanding. Since genetic diversity is linked to the processes of adaptation and extinction (Evans and Sheldon, 2008), high population-level genetic diversity and mean heterozygosity provide greater evolutionary potential for Philippine chickens. Increased population growth rate decreases the loss of genetic variation (Austerlitz et al., 1997); thus, diversity indices are a very essential foundation for potential genetic improvement and selection of species.

Previous fine-grained mtDNA phylogeographic study of chickens across the world revealed 13 divergent haplogroups (A–I and W–Z) (Liu et al., 2006; Miao et al., 2013) with the recent addition of haplogroup V (Huang et al., 2018). Haplogroups C and D are among the most diverse chicken haplogroups inhabiting East Asia and ISEA, respectively (Liu et al., 2006; Miao et al., 2013). They coalesced to form macrohaplogroup CDV approximately 8.1 kya with common ancestral motif at 306 nucleotide position, but haplogroup D diverge ∼4.4 kya harboring ancestral mutational motif at the 342 nucleotide position (Huang et al., 2018). Most RJF subspecies and their descendants are classified in haplogroup D, which is widely observed in the continental subclade, while a few are represented in the island clade (Liu et al., 2006).

This present work investigated the phylogeography of Philippine chickens and their possible dispersal to the Pacific. Phylogenetic analysis and MJ network revealed four distinct maternal haplogroups (A, B, D, and E) of Philippine chickens, with a predominant haplogroup D throughout the population. This confirms previous genetic evidence that haplogroup D is the maternal lineage largely concentrated in the ISEA–Pacific region (Liu et al., 2006; Miao et al., 2013) and distinctively traced as a specific signature for the Pacific sequence motif potentially found in the Philippines (Thomson et al., 2014). Both analysis from the complete and HVR mtDNA D-loop region of Philippine chickens, together with Indonesian and Pacific chickens, formed two subclades within subhaplogroup D1. The divergence pattern of Philippine–Pacific subclade harbored two mutational diagnostic motifs C296T and G686A, while undetected in the Philippine–Indonesian subclade concordant to the genealogical mitogenome classification reported by Huang et al. (2018) although using a few samples of Philippine chickens in the previous report. The newfound basal position patterns of Philippine RJFs in subhaplogroup D2 are grouped together with one of the early recorded Chinese gamecocks – Tulufan and the oldest Jidori-type breed in Japan – the Tosa-Jidori. The ancestral origin of Tosa-Jidori is suggested from the ISEA (Oka et al., 2007), while the ancestral origin of haplogroup D Tulufan gamecock in Northwest China is ambiguous because its diversity of distribution is mostly concentrated in haplogroups A and C (Miao et al., 2013), and independent admixture among gamecock breeds is evident (Luo et al., 2020). However, recent reports using whole-genome sequences showed a possible contribution of the local RJF subspecies or the earlier admixed domestic lineages from Yunnan Province, China, and MSEA (Luo et al., 2020; Wang et al., 2020). To date, the geographical distribution of subhaplogroup D2 is still unclear due to limited representation from other chicken populations across Southeast Asia. On the other hand, subhaplogroup D3 has geographical distribution in East China (Miao et al., 2013), South China, and Thailand (Huang et al., 2018), which likely suggests earlier introduction pattern to the Philippines from Indochina via early human migration movement (i.e., Negrito or First Sundaland people) around the Holocene period (Jinam et al., 2012; Lipson et al., 2014). The identified five haplotypes (*n* = 31) of Philippine NCs assigned to haplogroup E are believed to be the result of interbreeding between present-day chickens and commercial or show breeds. This haplogroup is widely represented in European domestic chickens and commercial lines with distinct haplotypes dispersed in the Middle Eastern and Indian subcontinents (Liu et al., 2006). This study also found two haplotypes in haplogroup A (*n* = 5) and four haplotypes in haplogroup B (*n* = 7), which are believed to have been introduced from neighboring countries including South and East China, Japan, and some countries in the MSEA (Liu et al., 2006; Oka et al., 2007).

Although Neolithic archeological records of Philippine chickens are still enigmatic (Piper, 2017), mtDNA evidence of Philippine–Pacific subclade provided strong inference for the Philippine origin of Pacific chickens, especially when both ancient and modern Pacific haplotype D (Polynesian motif) chickens (Thomson et al., 2014) clustered along with the Philippine chickens forming a subclade. The antiquity of the ancestral Polynesian haplotype previously described by Thomson et al. (2014) has been confirmed by its identification in Lapita contexts in Vanuatu (Petchey et al., 2015), which likely suggests an initial pattern of gene flow in the Melanesian populations before reaching Polynesia. The absence of Indonesian chicken sequences grouped with the ancestral Polynesian chicken motif (Thomson et al., 2014) and Philippine–Pacific subclade suggests a possible direct introduction from the Philippines. The most probable dispersal processes of Philippine chickens that might have contributed to the genetic characteristics of the Pacific chickens reflect the movement of the Austronesian speakers or the “out of Taiwan” migration model (Bellwood, 2007; Hung et al., 2011). This follows the Malayo–Polynesian dispersal in the Philippines about 2,200 BCE, and their continuous movement eastward through North Maluku to Island Melanesia before reaching Remote Oceania (Bellwood, 2007; Piper, 2017). In view of linguistic evidence, the Proto–Malayo–Polynesian term for domestic chickens appears to be widely recognized as far as Remote Oceania while distantly acquainted with the Proto-Austronesian speakers (Blust, 1995; Piper, 2017). Both genome-wide and mitogenome analysis of Austronesian speakers support an eastward movement harboring substantial aboriginal Taiwan-related ancestry approximately 4.4 kya (Lipson et al., 2014; Soares et al., 2016). However, Taiwanese indigenous chickens (e.g., Ju-Chi) and gamecock (Hua-Tung) do not exhibit haplotypes patterned for ISEA signatures; instead, they were influenced mainly from Chinese haplotypes and populations introgressed from the Indian subcontinent (Chang et al., 2012). This likely suggests that Austronesian speakers did not carry with them chicken species during their movement to the Philippines, but potentially took hold of earlier domestic lineages upon expanding eastward.

The result of our analysis indicated a very low genetic differentiation among chicken populations in the ISEA and the Pacific. High genetic closeness (*F*_ST_ < 0.2) was observed between Philippine and Pacific chickens (*p* = 0.001) than between Indonesian and Pacific chickens, which suggests little or no genetic difference among the former populations. This weak genetic structure is confirmed by the AMOVA analysis with 69.61% within-population variance (*p* = 0.000) for the Philippine–Pacific group, rejecting geographical-based isolation. This potentially reflects regional translocation from the Philippines going eastward to the Pacific by Austronesian speakers around <3,000 years ago (Soares et al., 2016). Support for the Philippine chicken expansion was provided by the unimodal mismatch distribution observed in the Philippine RJFs and Philippine NCs classified in haplogroup D. Conversely, Indonesian chickens and MSEA chickens appeared to be statistically unfit to satisfy the population growth model with large fractions of zero difference in the pairwise differences and with observed ragged mismatch distribution despite having high population diversity (Ray et al., 2003). This indicates that the population had undergone stability and/or population subdivision (Slatkin and Hudson, 1991). Philippine–Pacific subclade especially Pacific populations exhibited a star-like gene genealogy with more singleton sites (low frequency variants) and long terminal branches, characteristics that populations had undergone recent population growth across Oceania (Rogers and Harpending, 1992; Harpending et al., 1998).

It has been argued that the sudden demographic expansion model and raggedness statistics have limitations in detecting population expansion and estimation for demographic parameters (Rogers and Harpending, 1992; Harpending, 1994; Ramos-Onsins and Rozas, 2002). Therefore, we substantiated Tajima’s *D* and Fu’s *Fs* statistics to further infer possible population growth. In this work, we ruled out past population expansion signatures of Philippine RJFs and NCs (haplogroup D), which validated our previous analysis inferring the contributions of Philippine chickens to the genetic characteristics of the Pacific chickens. The negative and significant Fu’s *Fs* statistical test (Fu, 1997) provided strong evidence for the past population growth of Philippine chickens (haplogroup D) in the ISEA. Interestingly, the BSP analysis indicated demographic expansion of the Philippine chickens predating the recovered ancient DNA samples of Pacific chickens in the Anatoloa site, Niue Island, and the Anakena site, Rapa Nui (∼1,200–600 BP) (Thomson et al., 2014). This finding corroborated the eastward expansion of the Austronesian speakers from the Philippines before reaching the Pacific region. Overall, the Philippine–Pacific subclade is congruent with the evidence of increased maternal effective population size of Philippine chickens, while concordant with the demographic signals imprinted in DNA genealogies and timing of introduction brought by human dispersal (Figures 4, 5).

Estimates of genetic diversity, phylogeography, and population structure of Philippine chickens obtained in this study are characterized by a high level of genetic variability, especially influenced by the chicken populations identified as the haplogroup signatures for Philippine chickens. Although Philippine RJFs appear to have a fairly diverse population, flexibility for conservation efforts must not be neglected (Evans and Sheldon, 2008). An important direction for future work is to increase the density of sample populations from identified localized chicken breeds within the Philippines and from neighboring countries.

## Conclusion

This study provides an in-depth understanding of the matrilineal phylogeny, genetic diversity, and population dynamics of Philippine chickens. This explains the genetic relatedness of Philippine chickens with other chicken populations widespread in ISEA, especially the Philippine contribution to the genetic characteristics of Pacific chickens. The significant low genetic differentiation of Philippine chickens and Pacific chickens indicate a high level of gene flow among these chicken populations, which are mainly impacted by human-assisted movement around 3,500–3,000 years ago.

This study provides essential genetic information of these indigenous poultry resources for conservation efforts and that these data serve as a baseline for monitoring to avoid further loss of genetic diversity. This asserts great potential for genetic improvement and selection of valuable traits for developing sustainable chicken production systems in the Philippines.

## Data Availability Statement

The datasets presented in this study can be found in online repositories. The names of the repository/repositories and accession number(s) can be found in the article/Supplementary Material.

## Ethics Statement

The animal study was reviewed and approved by the Laboratory of Animal Genetics, Hiroshima University (015A170426). Animal owners personally consented the inclusion of their animals (native chickens) in the study.

## Author Contributions

CG, PD, DE, and MM conceptualized the study. CG and MM were in charge of the data curation. CG, MM, and MN developed the methodology and were in charge of the software. CG and MN performed the formal analysis. CG wrote and prepared the original draft. CG, MN, DE, and PD wrote, reviewed, and edited the manuscript. MN acquired the funding. All authors have read and agreed to the published version of the manuscript.

## Conflict of Interest

The authors declare that the research was conducted in the absence of any commercial or financial relationships that could be construed as a potential conflict of interest.

## Footnotes

1 http://genestudio.com/

## Supplementary Material

Supplementary Figure 1 Maximum likelihood (ML) phylogenetic tree showing four haplogroup classifications (predominant haplogroup D) of Philippine chickens and different classifications from other neighboring countries. Node labels correspond to bootstrap support values evaluated with 1,000 ultrafast bootstrap replicates in IQ-TREE. The scale bar (0.007) indicates the genetic distance (substitution per site).

Supplementary Figure 2 Median-joining (MJ) network of mitochondrial DNA (mtDNA) D-loop Hypervariable region (HVR) illustrating the genealogical relationships of chickens from Philippines (green), Indonesia (red), and Pacific (yellow) using all observed haplogroup D haplotypes (Supplementary Table 3). Genetic distance between Philippine–Pacific sub-clade and Philippine–Indonesian sub-clade are indicated by the length of branch corresponding for mutational positions. The area of each circle is proportional to the frequency of the corresponding haplotypes.

Supplementary Figure 3 Principal coordinate analysis (PCoA) plots of the population pairwise inter-haplotypic distance matrix for chicken populations in the islands of Southeast Asia (ISEA) and mainland Southeast Asia (MSEA) at complete D-loop region. Populations are assigned the following colors (Green: Philippine RJFs and native chickens (NCs); Yellow: Pacific chickens; Red: Indonesian chickens; Blue: MSEA chickens and other haplogroups).
