# Supplementary figures and images for: Population Genetic Structure and Contribution of Philippine Chickens to the Pacific Chicken Diversity Inferred From Mitochondrial DNA
Source: Front Genet. 2021 Jul 22;12:698401. doi: 10.3389/fgene.2021.698401 (PMC8340678; doi:10.3389/fgene.2021.698401)

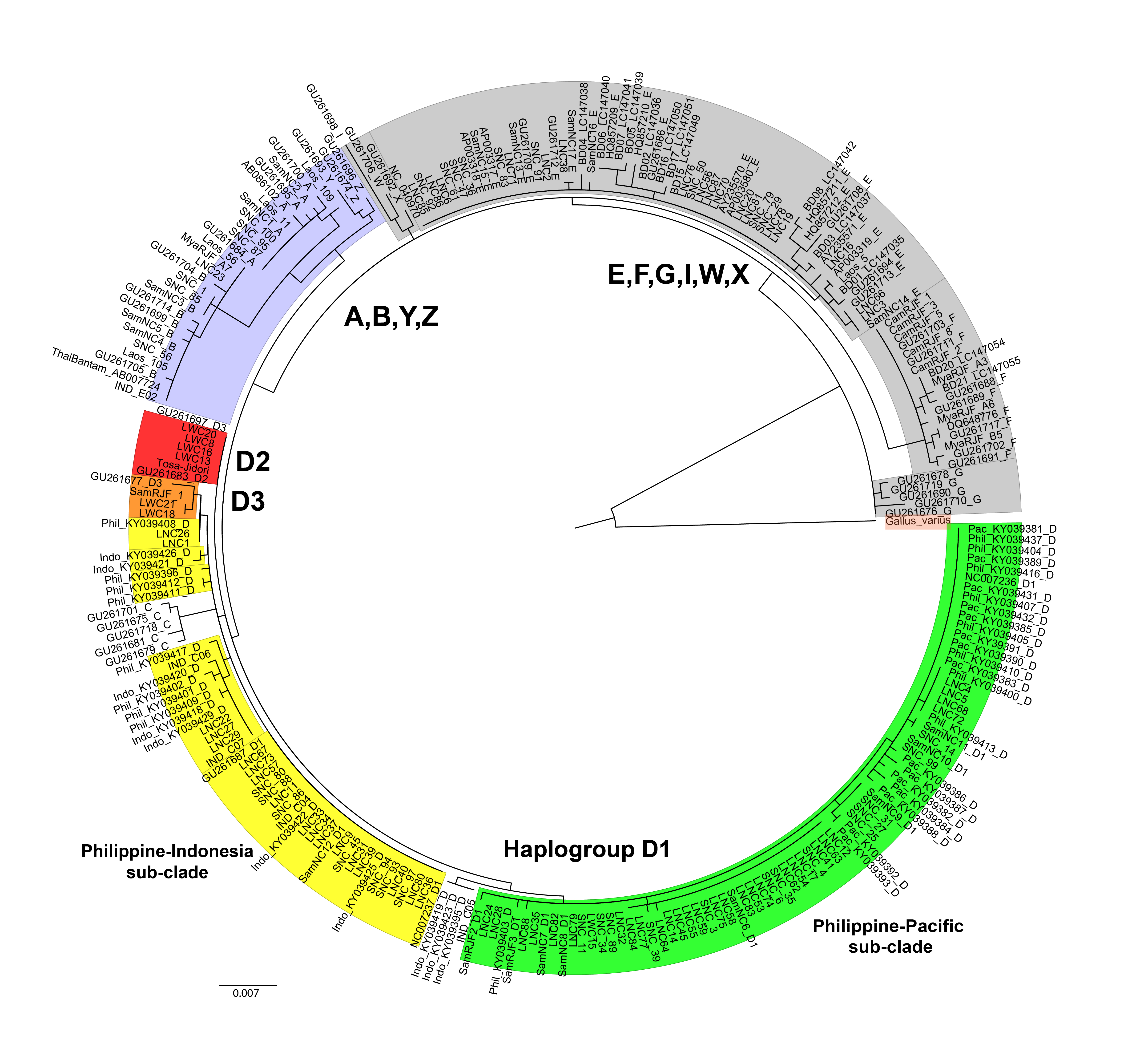

Supplement: Supplementary Figure 1 — Maximum likelihood (ML) phylogenetic tree showing four haplogroup classifications (predominant haplogroup D) of Philippine chickens and different classifications from other neighboring countries. Node labels correspond to bootstrap support values evaluated with 1,000 ultrafast bootstrap replicates in IQ-TREE. The scale bar (0.007) indicates the genetic distance (substitution per site). [file Image_1.JPEG]

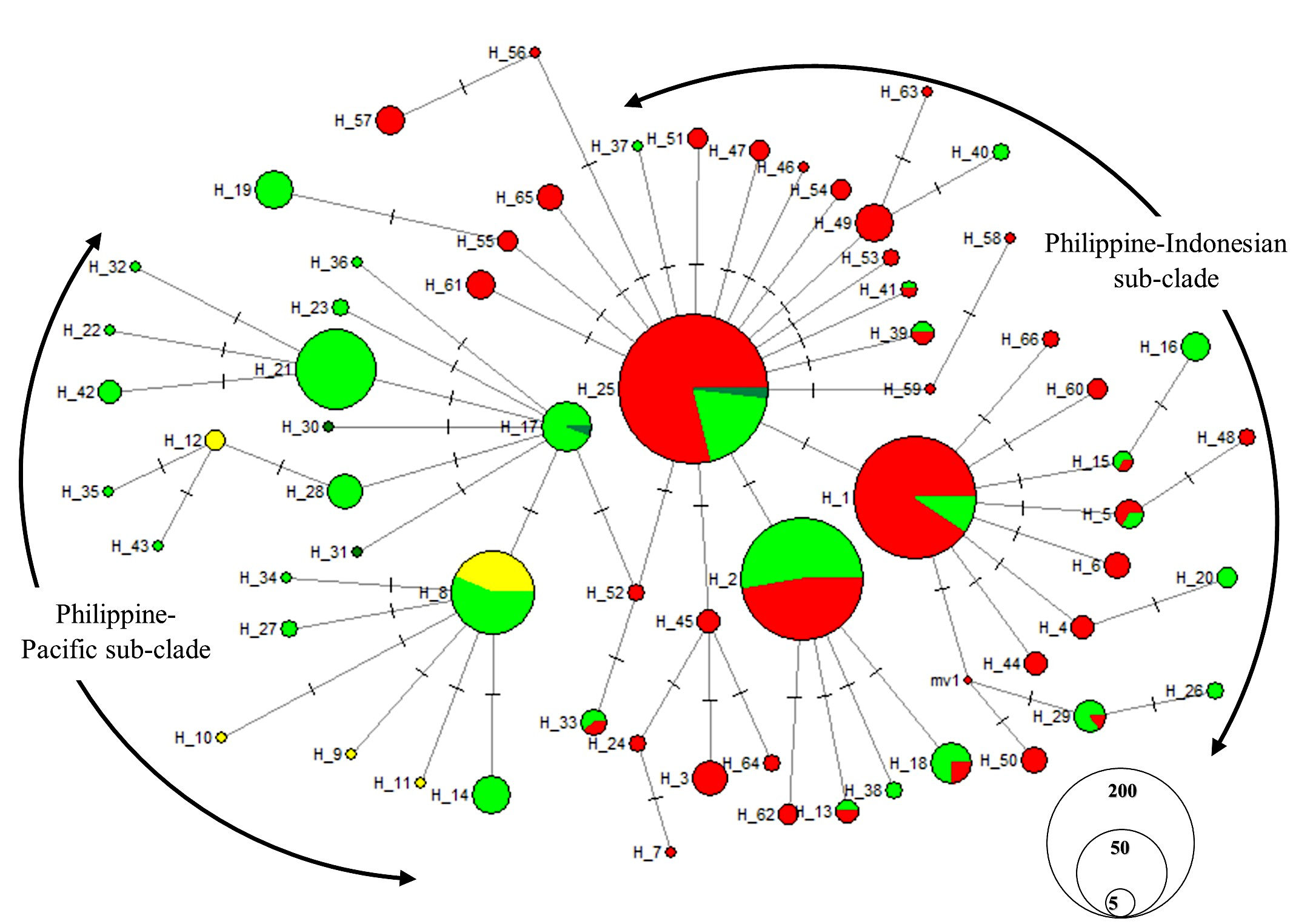

Supplement: Supplementary Figure 2 — Median-joining (MJ) network of mitochondrial DNA (mtDNA) D-loop Hypervariable region (HVR) illustrating the genealogical relationships of chickens from Philippines (green), Indonesia (red), and Pacific (yellow) using all observed haplogroup D haplotypes (Supplementary Table 3). Genetic distance between Philippine–Pacific sub-clade and Philippine–Indonesian sub-clade are indicated by the length of branch corresponding for mutational positions. The area of each circle is proportional to the frequency of the corresponding haplotypes. [file Image_2.JPEG]

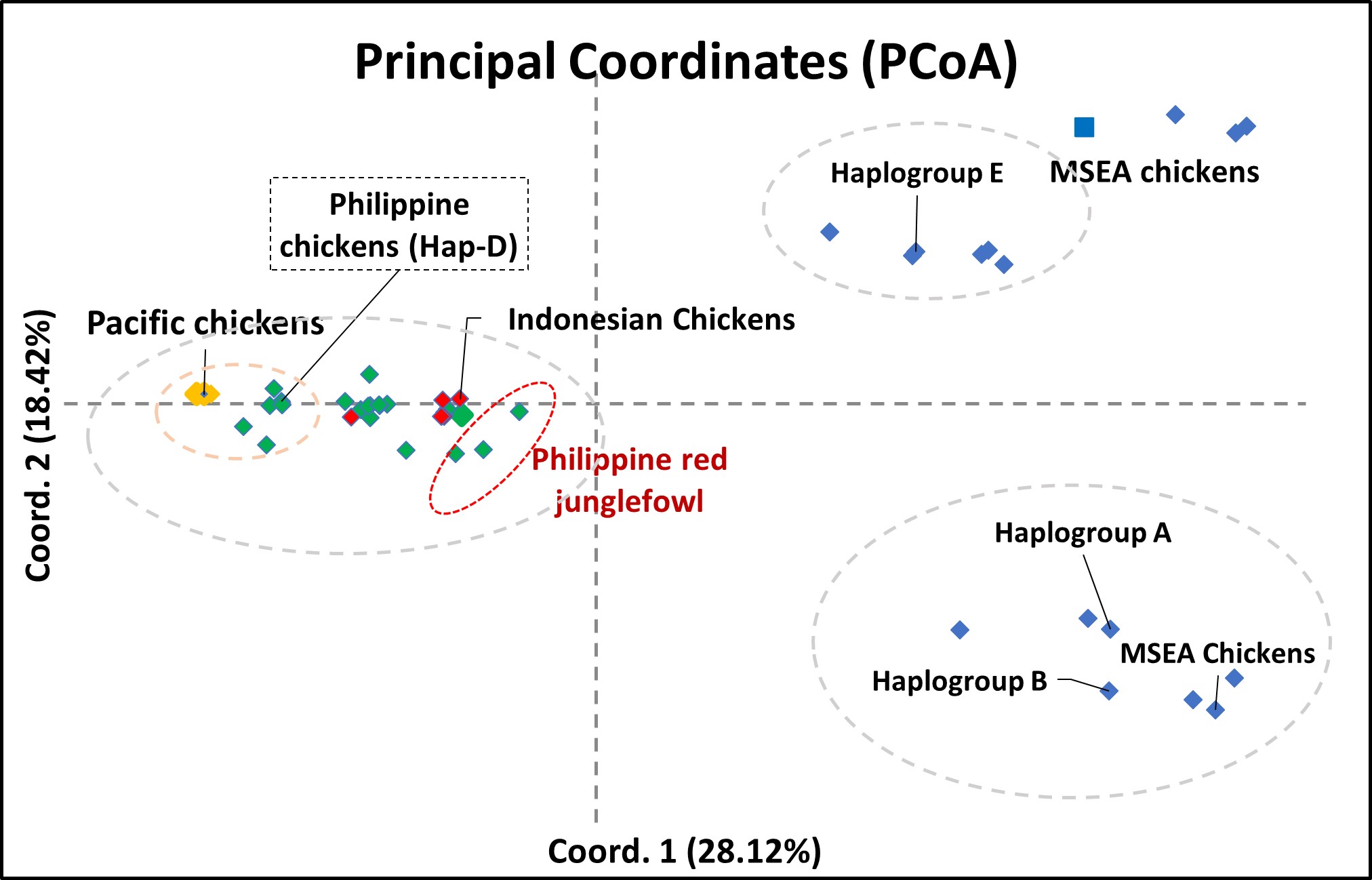

Supplement: Supplementary Figure 3 — Principal coordinate analysis (PCoA) plots of the population pairwise inter-haplotypic distance matrix for chicken populations in the islands of Southeast Asia (ISEA) and mainland Southeast Asia (MSEA) at complete D-loop region. Populations are assigned the following colors (Green: Philippine RJFs and native chickens (NCs); Yellow: Pacific chickens; Red: Indonesian chickens; Blue: MSEA chickens and other haplogroups). [file Image_3.JPEG]
